# A Statistical Journey through the Topological Determinants of the β2 Adrenergic Receptor Dynamics

**DOI:** 10.3390/e24070998

**Published:** 2022-07-19

**Authors:** Luisa Di Paola, Humanath Poudel, Mauro Parise, Alessandro Giuliani, David M. Leitner

**Affiliations:** 1 Unit of Chemical-Physics Fundamentals in Chemical Engineering, Department of Engineering, Università Campus Bio-Medico di Roma, Via Álvaro del Portillo 21, 00128 Rome, Italy; 2 Department of Chemistry, University of Nevada, Reno, NV 89557, USA; hpoudel@unr.edu; 3 Unit of Electrotechnics, Department of Engineering, Università Campus Bio-Medico di Roma, Via Álvaro del Portillo 21, 00128 Rome, Italy; m.parise@unicampus.it; 4 Environment and Health Department, Istituto Superiore di Sanità, 00161 Rome, Italy; alessandro.giuliani@iss.it

**Keywords:** GPCRs, β2 adrenergic receptor, allosteric switch, Protein Contact Networks, dynamical cross-correlation matrix, Multivariate Statistical Analysis

## Abstract

Activation of G-protein-coupled receptors (GPCRs) is mediated by molecular switches throughout the transmembrane region of the receptor. In this work, we continued along the path of a previous computational study wherein energy transport in the β2 Adrenergic Receptor (β2-AR) was examined and allosteric switches were identified in the molecular structure through the reorganization of energy transport networks during activation. In this work, we further investigated the allosteric properties of β2-AR, using Protein Contact Networks (PCNs). In this paper, we report an extensive statistical analysis of the topological and structural properties of β2-AR along its molecular dynamics trajectory to identify the activation pattern of this molecular system. The results show a distinct character to the activation that both helps to understand the allosteric switching previously identified and confirms the relevance of the network formalism to uncover relevant functional features of protein molecules.

## 1. Introduction

The allosteric character of membrane proteins has been an important topic for some time [1,2,3]. Molecular dynamics (MD) simulations of protein–lipid layer systems provide useful information for understanding the constrained dynamics of membrane proteins and how they communicate with the environment inside and outside the cell [1,4,5]; this knowledge is an essential starting point for the discovery of drugs targeting membrane protein receptors [6].

G-protein-coupled receptors (GPCRs) are the membrane proteins that transmit responses from the exterior of the cell to the interior [7,8,9,10]. The structural and conformational details of proteins are important not only to provide the insights that are vital for signaling, but also to target the drugs in the specific regions where the allosteric communication is facile. The signal transduction in proteins is mediated via the intra-protein networks. For GPCRs, several network analyses of intra-protein networks, such as buried ionizable networks, energy transport networks, and conserved non-covalent networks, have been examined [1,2,3]. Recently, Tan et al. explored the druggability of target proteins in terms of allosteric site identification for allosteric drugs, adopting rhodopsin, a G-protein-coupled receptor present in the rod cells of the retina as a model [11]: they applied a network approach to identify allosteric spots in the GPCR.

The present study extended the analysis of intra-protein networks in terms of Protein Contact Networks (PCNs) in GPCRs and compared the results with the recent results of energy transport networks for the same protein system [1,2,3].

Each GPCR shares a similar architecture composed of seven transmembrane helices, in which each helix is connected to another either by extracellular or intracellular loops. The extracellular side consists of a ligand-binding pocket, where the agonist or antagonist ligands bind, and the cytoplasmic region consists of the G-protein binding site. The GPCRs activate when an agonist binds to the orthosteric site.

The β2 adrenergic receptor (β2-AR) is a rhodopsin-like class A GPCR, an important drug target for its central role in bronchodilation. Moreover, β2-AR is a neurotransmitter receptor and is a potential drug target for asthma and cardiac disease. It is also a potential target for the obesity treatment [7]. There are several intersections of residue segments, known as motifs, that are found to be conserved in all class A GPCRs.

**Figure 1:**
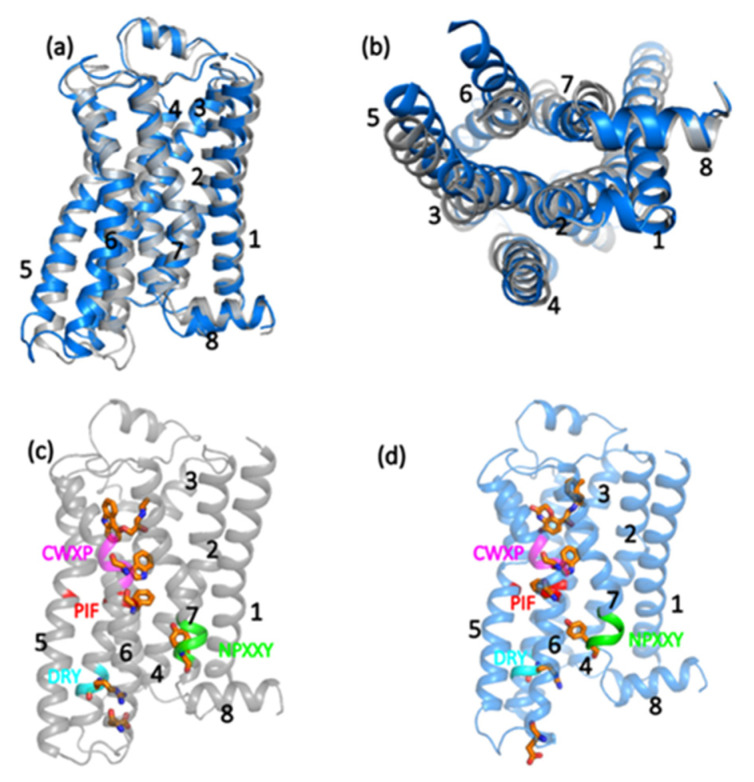
(**a**) The side view of the β2-AR. The active state (marine) is overlayed on the inactive state (gray). (**b**) The cytoplasmic view of the β2-AR depicts the opening of TM helix 5 and 6 outward in the active state compared to the inactive state. The conserved residues (motif regions) and molecular switches are shown for both active (**c**) and inactive (**d**) states. A distinct color, which is the same for both inactive and active states, is used to indicate each motif region. The stick representation is used to show the ligands, Tyr266 in NPXXY, Phe222 in PIF, Trp226 in CWXP, and the ionic lock (Arg103-Glu208) between TM3 and TM6. All structures are taken from the last frame of the 100 ns simulations for both states, in which the inactive state was simulated from PDB:2RH1, and the active state was simulated from the PDB:3P0G. The numbers in the figure label the 8 helices of the protein.

The structural comparison of the active and inactive structures of β2-AR is shown in Figure 1. Figure 1a shows the active state structure (marine) overlayed with the inactive state (gray). Figure 1b depicts the same overlayed comparison of the inactive and active states from the cytoplasmic point of view. The transmembrane helix 6, TM6, of the active state is shifted outward compared to the position in the inactive state. Similarly, a minor outward shift of TM5 and an inward shift of TM7 are also observed in the active state compared to the inactive state.

Figure 1c,d depicts the comparison of the molecular switches of β2-AR. A distinct color, which is the same for both inactive and active states, is used to indicate each motif. The stick representation highlights the ligands. The antagonist ligand (Carazolol C_18_H_22_N_2_O_2_) is attached to the inactive state in Figure 1c, and the agonist ligand BI-167107 (C_21_H_26_N_2_O_4_) is attached to the active state in Figure 1d. The switch residues Tyr266 in NPXXY, Phe222 in PIF, Trp226 in CWXP, and the ionic lock (Arg103-Glu208) between TM3-TM6 are also shown. The DRY (Asp-Arg-Tyr) motif is involved in the binding of the G-protein in the active state in the cytoplasmic region of TM3, where Arg acts as a switch forming an ionic lock with Glu208 of TM6. This lock is disrupted in the activation to promote the G-protein binding.

The CWXP (Cys-Trp-X-Pro, where x indicates a generic residue) motif is located at the bottom of the ligand-binding pocket in TM6, where Trp acts as a molecular switch. The Trp toggles in the active state, closing the ligand-binding pocket. The Tyr266 is, in turn, a molecular switch in the NPXXY (Asn-Pro-X-X-Tyr) motif in TM7, which rotates between different conformations. The PIF (Pro-Ile-Phe) motif expands in TM4, TM5, and TM6, where Phe reorients its side chain between conformations upon activation.

In a previous work [12], we studied energy transport networks and their reorganization with activation by an agonist, characterizing allosteric switches in charge of the allosteric signal transmission; the identification of the organizing centers of allosteric modulation of the β2-AR adrenergic receptors plays a relevant role in the drug discovery for these molecular systems.

This work presents a novel perspective on the β2-AR molecular system and its dynamics through the application of the Protein Contact Networks (PCNs [13,14]) methodology and the identification of network descriptors that aptly describe the allosteric transmission mechanism and identify the corresponding switches [15,16]. Additionally, the Multivariate Statistical Analysis (MVA [17,18]) of the molecular dynamics simulations of both active and inactive forms allow us to shed light on the different dynamical behavior of the β2-AR active and inactive forms, defining a sort of “activation fingerprint” based on concerted motions of residues.

Eventually, the MVA of the structural and network properties is a further proof-of-concept of the relationship between the protein structure, network topology, and protein function, being the PCN’s the necessary gap to explain in a detailed way the structure–function relationship in the β2-AR system and for protein molecular systems, more generally.

## 2. Materials and Methods

### 2.1. Molecular Dynamics Simulations

We simulated the β2 adrenergic receptor (β2-AR) in both inactive and active states. The simulations were set up by taking the coordinates from the protein data bank ID 3P0G [19] for the active state and 2RH1 [20] for the inactive state. This paper aims to compare the results of Protein Contact Networks (PCNs) to the results of Energy Transport Networks (EENs) of Reference [12]. To compare the analysis within the same systems and for consistency in comparison, the same crystal structures used in Reference [12] were chosen in this study. The missing residues of the intracellular loop of the active state were modeled by using Modeller version 9.23 [21]. Similarly, the missing residues of the inactive state were modeled by using the same program after omitting the T4-lysozyme chimera.

The initial simulation boxes were set up in the CHARM-GUI [22] online interface, which consists of 150 1-palmitoyl-2-oleoyl-sn-glycerol-3-phosphocholine (POPC) lipid molecules and about 6000 water molecules. The neutralization was performed by adding Na^+^ and Cl^−^ ions with a final concentration of 0.15M NaCl. All simulations were carried out in the AMBER16 MD software package under periodic boundary conditions, using AMBER ff14SB [23] forcefield for proteins, Lipid17 [24] forcefield for lipids, and TIP3P [25] forcefield for water molecules.

The systems were energy minimized for a total of 20,000 steps, using the steepest descent method for the first 10,000 steps and conjugant gradient for the remaining. After minimization, the systems were heated to 300 K from 0.1 K for 1 ns and held at 300 K for an additional ns. The heating was performed in an NVT ensemble with a Berendsen thermostat [26]. All hydrogen-containing bonds were constrained by using the SHAKE algorithm. Followed by heating, the systems were equilibrated for 10 ns with positional restraints in protein backbone atoms with a force constant of 1 kcal/(mol Å^2^). Finally, both systems were simulated for 100 ns, with an integration time of 2 fs, in an NPT ensemble, to analyze the dynamics.

### 2.2. Protein Contact Networks

The Protein Contact Networks (PCNs) are built up starting from the structural information contained in the PDB files [27]. The Contact Network nodes are the single residues and an edge between two nodes (residues) if the distance between their alpha-carbons falls between 4 and 8 Å. The mathematical counterpart of the PCN is the adjacency matrix, whose generic element (referring to node pair *i*-*j*) is defined as follows:

(1) $$A_{ij}=\{\begin{array}{c} 1\;if\;a\;link\;exists\;\\ 0\;otherwise \end{array}$$

The node degree, $k_{i}$, corresponds to the number of links a single node participates in and is computed as the sum of elements of the *i*-th row (or column) of the adjacency matrix:

(2) $$k_{i}=\underset{j}{\sum }A_{ij}$$

Connected to the definition of node degree, the clustering coefficient is defined for each node as the fraction of connected nodes that are also connected.

The shortest path matrix represents the connection map for the network; its generic element, $sp_{ij}$, is the shortest path (i.e., the minimum number of links in the PCN) connecting a pair of residues.

The betweenness centrality is defined as the shortest path’s matrix for a node and communicates to the other nodes in the network with their specific role in transmitting signals throughout the protein molecular structure; the generic value for the *i*-th residue $btw_{i}$ is the number of shortest paths passing by this node in the network. We computed the betweenness centrality by using the Kintali algorithm [28]. The betweenness centrality computed in structural network modeling has been largely demonstrated to address single nodes and their role in the signal transmission throughout biomacromolecules [27].

The closeness centrality is another centrality descriptor which relies on the shortest path. It is defined for each node as the inverse of the fairness of the node, which is the sum of all its shortest paths.

Finally, we computed the Jaccard similarity coefficient to assess the network similarities for the two forms PCNs and verify the extent of the rewiring upon activation; the coefficient is defined upon the two PCNs adjacency matrix as follows:

(3) $$Jacc=\frac{M_{11}}{M_{10}+M_{01}+M_{11}}$$

where $M_{11}$ is the number of contacts shared by the two matrices (networks); $M_{10}$ the number of contacts present in the first form, but not in the first; and $M_{01}$ the number of contacts present in the second form, but not in the first.

We computed the PCNs and relative descriptors through a purposed software [29].

### 2.3. Statistical Analysis of Molecular Dynamics Simulations

We applied a Multivariate Statistical Analysis approach to the molecular dynamics simulations to have a general perspective on protein dynamics. All the analyses follow a data-driven paradigm with no a priori assumption. We performed two different approaches, which are thoroughly described below.

*Canonical Analysis of Motion*

We analyzed data obtained from the molecular dynamics simulations in terms of a Canonical Analysis of Motion, which was applied to the whole set of frames of MD simulations for both conformations (active and inactive).

The Canonical Analysis of Motion is based on the displacement matrix, *D*, a m×p matrix, where *m* is the number of observations (single frames), and *p* is the number of variables (displacement of single residues). The displacement at a given time (frame), *j*, is computed as the Euclidean distance of the *i*-th residues at that time concerning their position at the time (*j* − 1); the first components of the displacement vectors are computed by using the position of the residue in the input PDB file as a reference. In other words, the displacement matrix columns are the time-ordered displacement vectors of the single residues along MD simulations.

Once computed, the displacement matrix, *D*, the generic element of the Dynamic Cross-Correlation (*DCC*) matrix, $DCC_{ij}$, reports the Pearson correlation coefficient between the displacement vectors of the *i*-th and *j*-th residues, and it is computed as follows:

(4) $$DCC\left(i,j\right)=\frac{<\Delta r_{i}\left(t\right)\cdot \Delta r_{j}\left(t\right)>}{\sqrt{<\|\Delta r_{i}\left(t\right){\|}^{2}>}\cdot \sqrt{<\|\Delta r_{j}\left(t\right){\|}^{2}>}}\;$$

where $r_{i}\left(t\right)$ represents the *i*-th residue coordinates (alpha-carbon) as a function of time, and $\Delta r_{i}\left(t\right)$ represents the residue displacement in the frame at *t*. Therefore, the generic element of *DDC* is the Pearson correlation coefficient between the displacement vector $\Delta r_{i}\left(t\right)$ of the *i*-th residue and $\Delta r_{j}\left(t\right)$ of the *j*-th residue.

High positive values in *DCC* correspond to residue pairs showing concerted motions, while values close to zero describe independent motions of residue pairs. In a recent work, we demonstrated that this method provides a faithful pictorial description of protein dynamics in a membrane protein in the light of concerted motions of protein regions [30].

*Multivariate Statistical Analysis of Global Molecular Properties in the Molecular Dynamics*

The Multivariate Statistical Analysis is a powerful tool for tracing the correlation patterns between the multivariate description of complex systems [31].

As aptly envisaged by Gorban et al. [32], the character of complex systems resides in the emergence of peculiar correlation structures among different features of the system at hand. In dynamics terms, the eigenvectors of the correlation matrix of the different features (corresponding to principal components) characterize a system trajectory in its phase space, generating an unbiased picture of the attractor states of the dynamics by the action of Takens’s theorem [33] This implies that any choice of *n* relevant features (with *n* > *p* being *p* the actual attractor dimension) computed along the trajectory can faithfully reproduce the attractor dynamics of the system at hand. This allows us to generate a faithful characterization of the system at hand, both in terms of concerted motions stemming from the presence of structurally relevant domains (Canonical Analysis of Motion) and in terms of correlated variance of global molecular properties.

Along this line of thought, we performed the correlation analysis of the whole set of variables computed for each MD frame, as listed in Table 1 (for a more detailed description of variables, see Supplementary Materials File S1 and Reference [34]).

It is worth noting that all of the abovementioned methodologies refer to second-order statistics (Pearson correlation), i.e., to methods relying on the particular disposition of statistical units. On the contrary, first-order statistics, such as mean and standard deviation of two X and Y variables, remain invariant by the independent shuffling of values across statistical units. Here, the arrangement of the statistical units (frames of the MD simulation) follows the time dimension; this implies that the emerging correlation structures are the image in light of the relationships holding among different features of the system at hand that make them covary in time. This correlation structure has nothing to do with the peculiar starting point of the simulation (that is largely arbitrary) but descends from the ‘mutual constraints’ among the considered variables.

This approach is, in other words, a classical ‘perturbation/relaxation’ experiment adopting the response of the system relaxing to its equilibrium state after a perturbation as a probe of its internal structure [35] and grounds on the analysis of transients. It is worth noting how order-independent statistics are unable to give a consistent picture of these out-of-equilibrium trajectories, while order-dependent (correlation-like) statistics can correctly identify the system dynamics [36]. In our case, this approach translates into the (apparently heretical) use of the initial and transient part of the dynamics when the system is out of equilibrium. While the arbitrary character of the starting point makes this transient/relaxation phase of no practical use for refined structural characterization, it is the most useful part of the dynamics for looking at the correlation structure. The increased range of variation (concerning the quasi-equilibrium phase) offered to the system during the transient phase allows us to highlight, thanks to the so-called ‘range restriction effect’ [37], relevant conditions that are otherwise impossible to discriminate by pure noise. Moreover, the virtual absence of any ‘real equilibrium’ condition in the living system makes this kind of analysis mandatory when dealing with biological problems [38].

## 3. Results

### 3.1. Analysis of Equilibrated Forms (Active and Inactive) of β2-AR

Here, we examine the results of Protein Contact Networks (PCNs) to identify the changes in the network upon activation of the β2-AR, and we compare the results of PCN with the results of Energy Exchange Networks (EENs) [39,40,41,42] computed for the same GPCR in a recently published computational paper [12].

**Figure 2:**
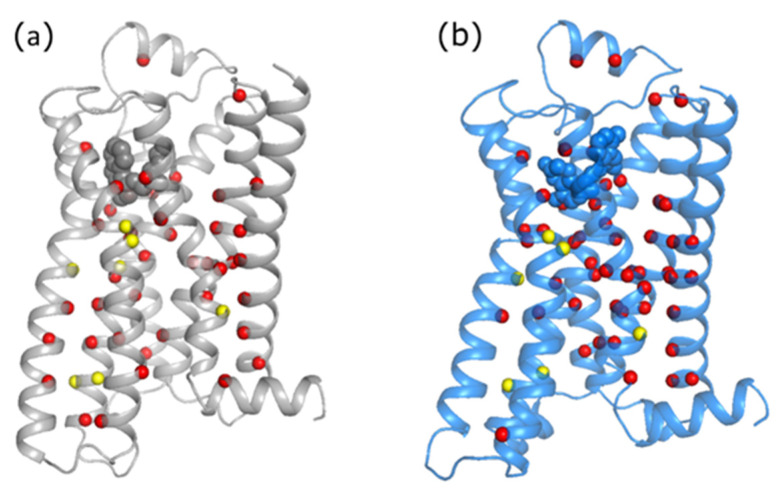
The residues that have node degrees larger than 10 ($k_{i}>10$) in the Protein Contact Network are shown. The inactive (**a**) and the active (**b**) states are shown in gray and marine colors, respectively. In yellow, the residues that fall in the motifs of β2-AR.

First, starting with the PCN node degree, *k_i_* (as defined in Equation (2)), we examine the similarities and differences in the inactive and active states of the β2-AR. The node degree, *k_i_*, corresponds to the number of edges (connections) an amino acid residue, *i*, shares with other residues in the network. In Figure 2, the nodes that have high values of degree ($k_{i}>10$) are shown for the inactive state (Figure 2a) and active state (Figure 2b). Figure 2a,b depicts the regions hosting the largest degree nodes in inactive and active states, respectively. These regions correspond to those below the ligand-binding pocket; CWXP motifs; and TM6, TM7, TM1, and TM4. The differences between inactive and active states are more evident on the lower side of TM5 and TM6. The opening of the TM helices, with TM5 and TM6 going outward, causes the loss of some interactions going from inactive to active state. The yellow color indicates the motif residues. The inactive state has more yellow residues, as is consistent with its ‘close’ structure. This is particularly relevant for the Ile93 of the PIF motif, which reorients upon activation (see Figure 1 for clarity).

**Figure 3:**
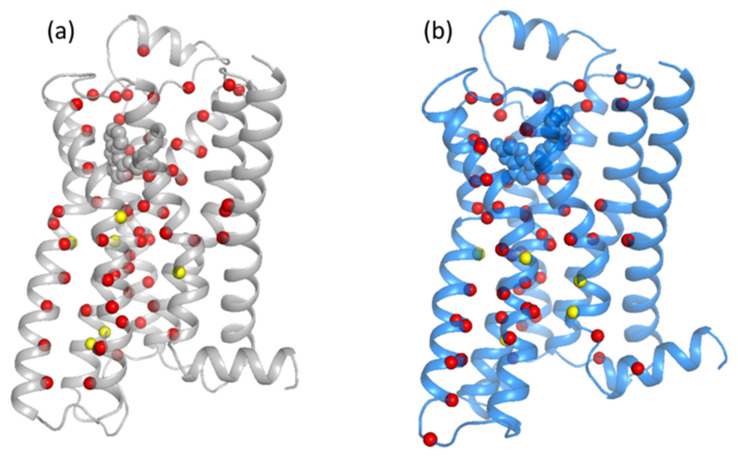
The residues that have betweenness centrality values larger than 1200 (btw>1200) in the Protein Contact Network are shown the (**a**) inactive state and (**b**) active state. The yellow is used to indicate the motif residues.

Now, we turn to the betweenness centrality, which measures the number of times a node lies in the shortest path between other nodes in the PCN. The residues that have significantly larger values of the betweenness centrality (*btw* > 1200) are shown as spheres in Figure 3. The yellow color indicates the residues of β2-AR that fall in the motifs. Both states depict a similar connection pattern: most of the high betweenness nodes are in the transmembrane helices, TM6, TM6, and TM7, and around the ligand-binding pocket. The main difference is seen in the TM7 of the active state, where the residues of the NPXXY motifs suggest a more continuous flow in the active state than in the inactive state.

As mentioned above, this work focuses on comparing the results of PCN to the results of EENs of Reference [12]; however, we further applied protein contact network analysis to the other crystal structures of β2-AR to validate how the method provides a similar result in other crystal structures of the same protein. Moreover, we applied the PCN to the fully activated GPCR attached with G-protein (PDB: 3SN6) and antagonist-bound inactive state (PDB 3NYA) (see Supplementary Materials). The results of PCN analysis in these two crystal structures are shown in Supplementary Figures S1 and S2 for the larger node degree and the larger betweenness centrality. Both Supplementary Figures S1 and S2 are very similar to Figure 1 and Figure 2 (which were computed from the last frame of the MD simulations of inactive, PDB:2RH1, and active PDB:3P0G, states). The regions observed in Supplementary Figures S1 and S2 are very similar to Figure 1 and Figure 2, respectively, for both active and inactive states.

**Figure 4:**
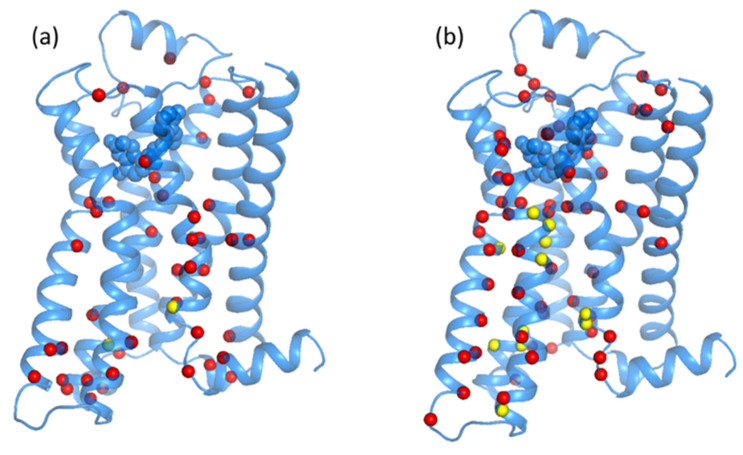
The highlighted residues correspond to the largest differences in degree ($\left|\Delta k_{i}\right|>1$) (**a**) and betweenness centrality ($\left|\Delta btw_{i}\right|>500$ ) (**b**).

A detailed comparison of the node degree and the centrality measures are presented in the difference map of Figure 4.

For the difference map, we computed the difference in degree: $∆k_{i}=k_{i}^{active}-k_{i}^{inactive}$. Figure 4a reports the map on the inactive state β2-AR ribbon structure of the largest $\Delta k_{i}$ (in absolute value larger than 1). The largest differences are in the cytoplasmic region where the opening of the structures occurs at the activation of the GPCR. Similarly, other changes are seen in the TM7, where the Tyr266 of the NPXXY motif reorients in the activation; other changes are also observed in the TM7.

Finally, we turn to the difference in the betweenness centrality for the residues of the β2-AR. We defined the difference in betweenness centrality as $∆btw_{i}=btw_{i}^{active}-btw_{i}^{inactive}$. Figure 4b reports the largest values of $∆btw_{i}$ (in absolute value larger than 500) on the β2-AR inactive state ribbon structure. Figure 4b highlights two major differences between the two states: (i) motif residues show the highest values of $\left|\Delta k_{i}\right|$ and (ii) prevalence of changes in the part of the structure going from the ligand-binding pocket to the cytoplasmic region of the β2-AR. Comparing the results of PCN to the results of the Energy Exchange Network computed in Reference [12], we note many similarities between Figure 4b, with the r∆EEN results stemming from energy flow simulations in the prior study [12]. The location of the residues in Figure 4b is similar to the results of r∆EEN following from the ligand-binding pocket to the cytoplasmic regions through TM helices 5, 6 and 7 and for Phe222 (the residue of the PIF motif), acting as a switch. The main purpose of the r∆EEN was to capture the interactions between the van der Waals interactions because they depicted significantly smaller rates of energy transfer between them compared to the polar and charged contacts [39,43].

Table 2 reports the overview of the global properties of the equilibrated forms of β2-AR.

The *MFD* is lower in the active form, pointing to a slightly looser structure, which is also characterized by a shift to a more spherical shape (slightly lower *As*). However, values in the range 2.5–2.7 are typical of proteins of this size [44]. The value of *D* affects energy transport dynamics in the protein [45,46], and the differences found here are consistent with the more robust energy transport observed for the active state compared to the inactive state of β2-AR [12].

The rewiring is quite high (30% as indicated by the Jaccard index), but this rewiring does not imply any relevant change in the average values of topological descriptors (*adeg*, *abtw*, *asp*, and *E*). This result highlights the high resilience of Protein Contact Networks in network dynamics [47].

### 3.2. Statistical Analysis of Molecular Dynamics Simulations of $\beta_{2}$-AR Forms (Active/Inactive)

*Canonical Analysis of Motion*

**Figure 5:**
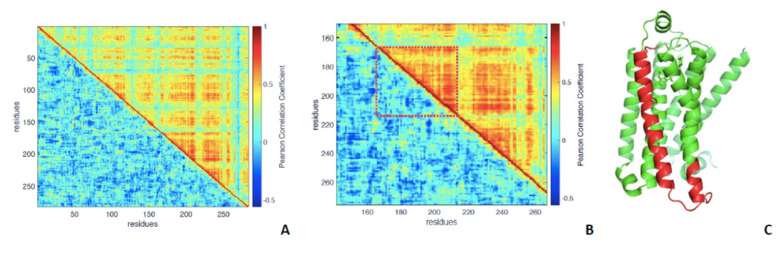
*DCC* heat maps for β2-AR: (**A**) inactive state is in the lower triangular matrix, active in the higher. (**B**) Magnification of a trait between residues 150–270; in the rectangle, the sequence trait in the residue range 167–213 is highlighted, showing high correlated motions in the active state (upper part, hotter colors, and visible patterns) against the inactive state, where the same trait shows poorly correlated motions (colder colors and no patterns). (**C**) As a visual reference, the trait 167–213 is shown in red in the ribbon representation of the active state.

Figure 5 is the heat map of the *DCC* matrix (see Section 2); Figure 1a shows a comparison of the *DCC* for active (upper triangular matrix) and inactive (lower triangular). The heat map visually guides to identify correlated motions, shown by hot colors, while cold colors point to a poor correlation of motions between residues.

As an example, Figure 5B shows a magnification of the trait 167–213 (in the dotted square), clearly showing the great difference in terms of the correlation of residue motions between the two forms: the upper part (active) is characterized by hot colors and visible patterns of correlations (red bands), while the lower, referring to the inactive form, is characterized by much colder colors and no visible patterns. Figure 1c shows the trait in red on the ribbon structure of the active form to serve as a visual reference.

The squared texture of the active state correlation matrix is the image in light of the emergence of dynamical ‘domains’ spanning the entire structure, interspersed by ‘independent motion’ (blue lines) residues.

It is evident that the active state is characterized by a higher correlation level than the inactive state, which means that the residues in the active state move in concerted motions with each other more than in the inactive state; the average correlation coefficient in *DCC* in the inactive state is 0.31, and in the active state, it is 0.71.

*Multivariate Statistical Analysis of the Molecular Dynamics*

**Figure 6:**
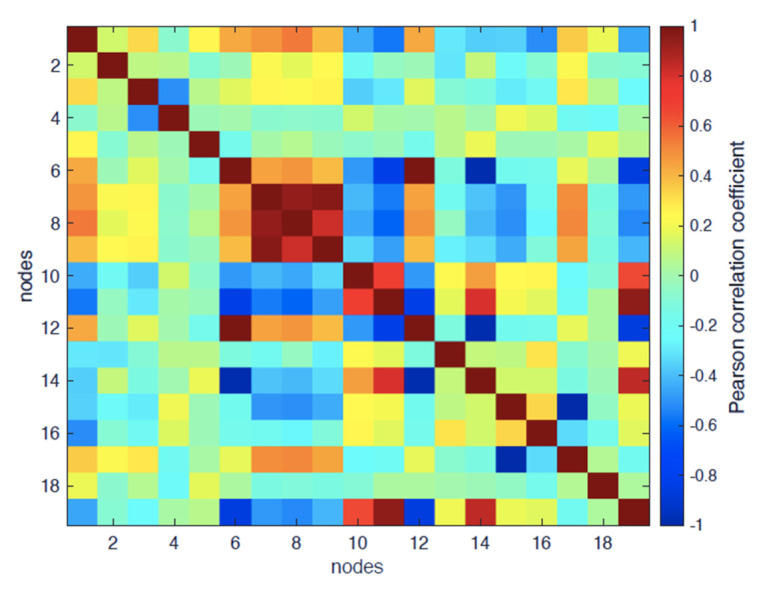
Pearson correlation map for the inactive form of the β2-AR.

Figure 6 reports the heat map of Pearson correlation between the variables in Table 1 for the inactive form of the β2-AR; some variables significantly linearly correlate with time, *corr*(*t*, *R_G_*) = 0.54, *corr*(*t*, *adeg*) = −0.56, *corr*(*t*, *Mfd*) = −0.52; that means that they show a trend to linear variation along the relaxation trajectory.

The variables describing the network topology can be divided into two categories:

1. Degree-based: *adeg* and *E* (considering that *corr*(*E*, *adeg*) = 0.95, meaning that *E* is practically overlapping with *adeg*);
2. Shortest-path-based: *abtw, aclose* and *asp*.

As expected according to the network theory, there is a strong correlation between *adeg* and *asp*: *corr*(*asp*, *adeg*) = −0.83, reporting the known fact that, at increasing degrees, the shortest path decreases (more efficient signal transmission at a distance due to the largest number of possible ‘shortcuts’ generated by the increasing number of edges).

As for purely structural variables, *R_G_*, as expected, strongly correlates with both *R_Gh_* and *R_Gp_* (0.94 and 0.96, respectively).

**Figure 7:**
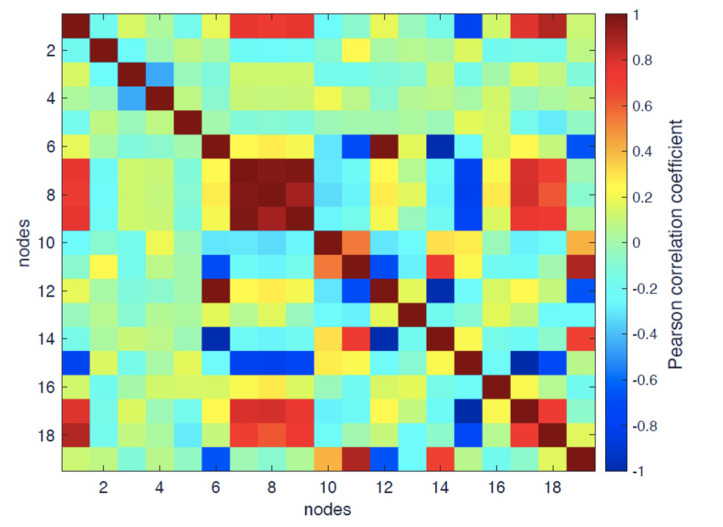
Pearson correlation map for the active form of the β2-AR.

Figure 7 shows the heat map of the Pearson correlation between variables in Table 2 for the active form. As for the inactive form, some variables significantly linearly correlate with time: *corr*(*t*, *R_G_*) = 0.54, *corr*(*E*, *adeg*) = −0.56, *corr*(*t*, *MFD*) −0.52.

We consider, as for the inactive form, the topological variables divided into two categories:

1. Degree-based: *adeg* and *E* (*corr*(*E*, *adeg*) = 0.95);
2. Shortest-path-based: *abtw*, *aclose*, and *asp*.

It is worth noting the decrease in the entity of the ‘obliged’ correlation between *adeg* and *asp*: *corr*(*asp*, *adeg*) = −0.69 for the correlation observed in the inactive state (−0.82); this implies that, in the active state, *asp* is modified by the concurring of other wiring structure modifications in addition to the simple connectivity changes.

As for purely structural variables, even in this case, *R_G_*, as expected, strongly correlates with both *R_Gh_* and *R_Gp_* (0.97 and 0.98, respectively); the two partial radiuses of gyration show a direct correlation of 0.82 consistently with the inactive case. *R_G_* correlates with both *AS* (0.72) and porosity (0.80).

Table 3 reports, for the inactive form, the best score for correlation coefficients in the canonical analysis of variables, as parted in the following: time $X_{1}=t$; topological variables $X_{2}=\left\{adeg,\;asp,\;abtw,\;aclose,\;acc,\;E\right\}$ and structural variables $X_{3}=\left\{R_{G},\;R_{Gh},\;R_{Gp},\;\rho ,\varepsilon ,AS,\;MFD\right\}.$

In the inactive form, collective topological ($X_{2}$) and structural ($X_{3}$) variables both have a good correlation with time ($X_{1}$), given that the structural changes along dynamics are evident at any level (shape, size, and intramolecular contacts network). Eventually, it is interesting to highlight a good general correlation (canonical) between topology and structure collective variables, *X*_2_ and *X*_3_.

Table 4 shows the best scores of correlation coefficients in the CCA for the active form.

In this case, the linear correlation between time ($X_{1}$) and structural descriptors ($X_{3}$) is stronger in the inactive state, 0.91, than the inactive state, 0.70, and weaker for topological ($X_{2}$, 0.54 vs. 0.72), and the cross-correlation between structural and topological variables (0.55 vs. 0.74) is weaker.

Finally, the application of the PCA allowed us to further clarify the role of variables in protein relaxation dynamics toward the attractor equilibrium state.

We limited ourselves to the first two PCs, reporting only the relevant (>|0.5|) loadings (Pearson correlation between variables and components) distribution.

In the case of the inactive state, we observed the following for the first two:

(a) PC1 (accounting for 37.2% of total variance): *t* = −0.67; *abtw* = −0.83, *R_G_* = −0.78, *R_Gh_* = −0.80, *R_Gp_* = −0.7; *acc* = −0.68, *adeg* = −0.88; *aclose* = 0.78; ρ = 0.5; *E* = 0.86; this component accounts for the relaxation dynamics (negative correlation with *t*), driving all listed topological and structural variables.
(b) PC2 (accounting for 14.4% of total variance): *aclose* = 0.59, *ρ* = −0.66; this component variance is not addressed by the linear trend toward the equilibrium state and points to time-invariant features of the structure.

In the case of the active state, the PCA loadings profile (for the first two components) is as follows:

(a) PC1 (accounting for 34.8% of total variance): *t =* −0.80; *abtw* = 0.56, *R_G_* = −0.89, *R_Gh_* = 0.89, *R_Gp_* = 0.85 *adeg* = −0.51; *asp* = 0.56, *aclose* = −0.54; ρ = −0.87; ε = 0.87, *AS* = 0.71; this component accounts mainly for the linear trend, driving all listed topological and structural variables;
(b) PC2 (scoring 11% of total variance): *abtw* = −0.74; *adeg* = 0.72, *aclose* = 0.76, *E* = 0.81; again, this variance is not addressed by relaxation dynamics.

The PCA results show a strong resemblance between the relaxation dynamics of inactive and active states, thus confirming, albeit indirectly, the robustness of the transient dynamics correlation analysis. It is worth noting the stricter correlation of PC1 with time in the case of the active state, which is consistent with the results of the Canonical Analysis of Motion.

## 4. Discussion

The results of the Protein Contact Networks analysis demonstrate the good compliance of the PCNs with the EENs method; namely we were able to find a good matching with the allosteric switches found with EENs in Reference [43] with residues endowed with high values of betweenness centrality. This finding is consistent with earlier work on the A_2A_ adenosine receptor, another rhodopsin-like GPCR, carried out by Hyeon and coworkers, who found that the betweenness centrality of a PCN was a good identifier of allosteric switches [3]. The betweenness centrality has been already addressed in other works as a crucial topological descriptor of PCNs linked to the allosteric signal transmission [48]. Future work will need to consider more directly the role of water molecules in the transmembrane region that are known to contribute to allosteric regulation in GPCRs and play a central role in GPCR dynamics and activation [49].

According to the results of the correlation of motions, along with the MD simulations (see Figure 5), the two forms show distinct patterns, even though they show a very similar topology (see Topological Descriptors in Table 2): the active form, characterized by a lower average degree than for the inactive one, shows, in turn, a more extensive attitude to concerted motions of residues along the MD simulations than the inactive form.

Finally, the canonical correlation analysis of the MD simulations for both forms (see Table 2 and Table 3) showed that the collective variables describing PCNs topology and protein structure are in good correlation to each other (better in the inactive than in the active state) and distinctly to time (the best is structural in the active, and the worst is topological in the active state).

Recently, the structures of β2-AR small molecule allosteric ligands [50,51], positive allosteric modulators (PAMs), and negative allosteric modulators (NAMs) have been made available, providing new insights for allosteric signaling and the opportunities and advancement for allosteric drug design [52].

In the future, it is worthwhile to extend the PCN analysis for structures bound with the allosteric modulators to better understand the signaling through GPCRs. In addition, the Protein Contact Networks can be employed directly in the crystal structures, as applied here for the inactive state, PDB: 3NYA, and active state, PDB:3SN6; this method has the potential to analyze many structures of the whole GPCR family to identify the common signaling pathways, which would help to advance our understanding of cell signaling.

## 5. Conclusions

The good matching of the allosteric switches in the β2-AR system identified by protein contact networks with those previously found by energy transport networks is strong proof that the Protein Contact Networks provide a lean yet general framework to describe the structure–function relationship in protein molecular structures. Furthermore, similar observations in the networks from the MD simulations and crystal structures provide the broader application and efficiency of the method for analyzing the large range of crystal structures within the same receptors and the same class of GPCRs.

The coherence between the static (PCN) and dynamical (MD) approach provides valuable insight for membrane proteins (TRAF2 [17,18]) and opens the door to pharmacological applications [1,2,3].

Still more relevant, in our opinion, is the emergence of a self-consistent picture ‘keeping together’ the structure (with graph formalization acting as a sort of protein structural formula) description, the molecular dynamics, and the analysis of the relaxation transient toward equilibrium. These different views give support to each other, suggesting the existence of shared organizational principles allowing us to envisage the rise of an integrative biological complex system science.

## Figures and Tables

**Table 1:** Multivariate Statistical Analysis variables.

Symbol	Short Description
**Network Topology**	
*DBA*	Degree-based assortativity
*Dy*	Diadicity
*H*	Heterophilicity
*HBAKD*	Hydrophobicity-based assortativity
*abtw*	Average node betweenness centrality
*acc*	Average node clustering coefficient
*adeg*	Average node degree
*asp*	Average shortest path
*aclose*	Average node closeness centrality
*E*	Graph energy
**Molecular Structure**	
$R_{G}$	Radius of gyration
$R_{Gh}$	The radius of gyration of hydrophobic residues
$R_{Gp}$	The radius of gyration of polar residues
*corrHBKD*	Hydrophobic core probability
ρ	Mass density
*MFD*	Mass fractal dimension
ε	Porosity (void fraction)
*AS*	Asymmetry index

**Table 2:** Comparison of global properties of the equilibrated forms (inactive and active) of β2-AR.

	Inactive	Active
**Structural properties**		
*MFD*	2.70	2.52
*R_G_*, *Å*	10.07	10.20
*ε*	0.31	0.38
*AS*	0.53	0.46
*corrHb*	−0.12	−0.09
**Topological properties**		
*adeg*	7.27	7.50
*abtw*	842	816
*asp*	5.28	5.24
*E*	587.2	591.9
*Jacc*	0.703	

**Table 3:** Best scores of correlation coefficients as for canonical correlation for the inactive form.

	X_1_	X_2_	X_3_
*X_1_*	1	0.72	0.70
*X_2_*	0.72	1	0.74
*X_3_*	0.70	0.74	1

**Table 4:** Best scores of correlation coefficients as for canonical correlation for the active form.

	X_1_	X_2_	X_3_
*X*_1_	1	0.54	0.91
*X*_2_	0.54	1	0.55
*X*_3_	0.91	0.55	1

## Data Availability

Not applicable.

## Supplementary Materials

The following supporting information can be downloaded at https://www.mdpi.com/article/10.3390/e24070998/s1. Supplementary File S1: Full list of variables in Table 1; Figure S1: The residues that have a degree value larger than 10 (*k_i_* > 10) in the protein contact network are shown as spheres. Left: The inactive state PDB: 3NYA and right: active state PDB: 3SN6 are shown. In yellow the residues that belong the motifs of β2-AR are highlighted. Figure S2: The residues that have the betweenness centrality measure value larger than 1200 in the Protein Contact Networks analysis are shown as spheres. Left: The inactive state PDB: 3NYA and right: active state PDB: 3SN6 are shown. In yellow the residues that fall in the motifs of β2-AR are highlighted. References [53,54,55,56,57] are cited in the supplementary materials.
