# Supplementary material for: A Statistical Journey through the Topological Determinants of the β2 Adrenergic Receptor Dynamics
Source: Entropy (Basel). 2022 Jul 19;24(7):998. doi: 10.3390/e24070998 (PMC9318934; doi:10.3390/e24070998)
Supplement: Supplementary file 1 [file entropy-24-00998-s001.zip › entropy-1769652-supplementary.pdf]

Supplementary File S1

Full list of variables in Table 1

As follows, a complete description of variables listed in Table 1:

1. *t,* time: this variable is required to verify which other variable is varying during dynamics;
2. *DBA*, degree-based assortativity: this variable measures the probability that two connected nodes have also similar degrees [1];
3. *Dy*, dyadicity, represents the ratio between the number of edges connecting nodes with the same hydrophobicity (either negative or positive, according to the Kyte-Dolittle hydrophobicity scale[2]) over the number of those expected in a random distribution (with respect to hydrophobicity);
4. *H*, heterophilicity, it is the dual property of *Dy*, it is the ratio between edges connecting nodes with dissimilar hydrophobicity over the number in a random distribution with respect to hydrophobicity;
5. *HBA*, hydrophobicity-based assortativity: it measures the probability that two connected nodes have also similar hydrophobicity;
6. *abtw*, average betweenness: the average value of the aforementioned PCN betweenness centrality averaged over the number of nodes (residues);
7. R_G, radius of gyration: it is the radius of gyration of the protein molecular structure, defined as:

$$R_G = \sqrt{\frac{1}{2}\frac{\sum_{i=1}^{n} m_i r_i^2}{\sum_{i=1}^{n} m_i}} \qquad (5)$$

the sum is extended to all *n* residues;

8. R_{Gh}, radius of gyration of hydrophobic residues: as for the general definition, the sum refers only to hydrophobic residues;
9. R_{Gp}, radius of gyration of polar residues: as for the general definition, the sum refers only to polar residues;
10. *acc*, average clustering coefficient: it measures the average value of the above described clustering coefficient, averaged over the number *n* of nodes (residues);
11. *adeg*, average degree: it measures the average value of the aforementioned node degree, averaged over the number *n* of nodes (residues);

12. *asp*, average shortest path: it is the average value of the shortest paths connecting node pairs in the network, averaged over the whole number $n(n-1)/2$ of node pairs;
13. *corrHB*, hydrophobic core probability: it measures the probability of a residue to be placed with respect to the protein center of mass according to its hydrophobicity;
14. *aclose*, average closeness centrality: the average value of the above described closeness centrality, averaged over the number n of nodes (residues);
15. ρ, protein mass density: it measures the ratio between the protein mass and its volume, in units of g/mol Å^3;
16. *MFD*, mass fractal dimension: it is defined according to the Haussdorff scaling law [3]:

$$M \propto R^{MFD} \quad (6)$$

It is close to 3 for compact 3D objects, close to 1 for linear, rodlike shapes, while fractal dimensions close to 2 indicate characterize planar structures. Typically, the protein folding process generates shapes in between 2 and 3 MFD values [4].

17. ε, protein porosity: it is defined as the void fraction of the protein volume;
18. *AS*, the asymmetry index: it provides a quantitative description of the protein molecular shape [5]. When close to 0, the protein molecule is globular, close to 0.5 it is principally a plane molecule, whereas *AS* approaches to 1 for rodlike molecular proteins;
19. *E*, graph energy. It is a purely topological descriptor, which is computed as the sum of the absolute values of the adjacency matrix *A* eigenvalues. It is correlated to protein structural stability and has already been introduced to characterize the protein-protein interactions [6].

We applied three different statistical tools to the *m X p* matrix *M* (being *m=19* the number of variables, as above detailed, and *p* the number of frames we applied the analysis to) of the structural properties:

1. Correlation analysis: we computed the Pearson correlation analysis of the matrix *M* to highlight the correlation patterns between variables; we also applied the partial correlation between pair of variables, excluding the effect of a third, to determine the degree of association between the two variables (under the hypothesis of randomness), removing the effect of a third controlling variable. This metrics is particularly suited to reveal real mutual effects between variables in complex systems [7,8]; we'll use the following notation $corr(X,Y)_Z$, meaning "the partial correlation of the variables *X* and *Y* excluding the effect of variable *Z*".

2. Canonical Analysis: on the basis of results of correlation and Principal Component Analysis, it is possible to group variables into categories; for instance, as for the abovementioned variables, it is possible to identify a Topological Group, $X_2 = \{adeg, asp, abtw, aclose, acc, E\}$ and a Structural Group $X_3 = \{R_G, R_{Gh}, R_{Gp}, \rho, \varepsilon, AS, MFD\}$. The Canonical Analysis finds the linear combination within groups providing the pair of variables (X_1^{OPT}, X_2^{OPT}) which scores the highest Pearson Correlation Coefficient. We added as separate variable to this analysis the time: $X_1 = t$.
3. Principal Component Analysis (PCA): this methodology allows to transform the $M{=}m\,X\,p$ matrix of observations in a new set of orthogonal variables, the Principal Components (PCs), which are linear transformations of the original set $m\,X\,p$. Principal Components loadings are the Pearson correlation coefficients between the original variables and PCs.

Protein contact network analysis of β_2 adrenergic receptor

The Protein contact network analysis of crystal structures of β_2 adrenergic receptor in the active PDB: 3SN6 and inactive PDB: 3NYA states, respectively.

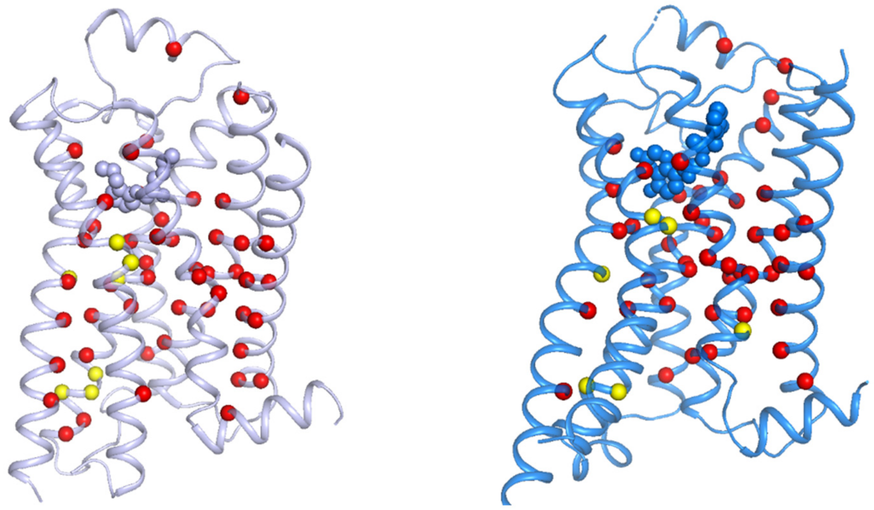

Figure S1. The residues that have a degree value larger than 10 ($k_i > 10$) in the protein contact network are shown as spheres. Left: The inactive state PDB: 3NYA and right: active state PDB: 3SN6 are shown. In yellow the residues that belong the motifs of β2-AR are highlighted.

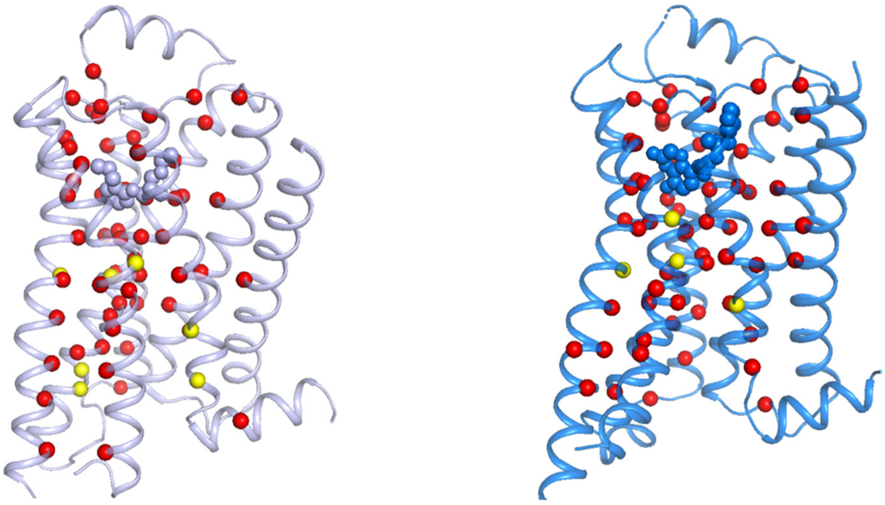

Figure S2. The residues that have the betweenness centrality measure value larger than 1200 in the Protein Contact Networks analysis are shown as spheres. Left: The inactive state PDB: 3NYA and right: active state PDB: 3SN6 are shown. In yellow the residues that fall in the motifs of β2-AR are highlighted.

REFERENCES

[1] M.E.J. Newman, Assortative Mixing in Networks, Physical Review Letters. 89 (2002). https://doi.org/10.1103/PhysRevLett.89.208701.

[2] J. Kyte, R.F. Doolittle, A simple method for displaying the hydropathic character of a protein, Journal of Molecular Biology. 157 (1982). https://doi.org/10.1016/0022-2836(82)90515-0.

[3] M.B. Enright, D.M. Leitner, Mass fractal dimension and the compactness of proteins, Physical Review E - Statistical, Nonlinear, and Soft Matter Physics. 71 (2005). https://doi.org/10.1103/PhysRevE.71.011912.

[4] L. di Paola, P. Paci, D. Santoni, M. de Ruvo, A. Giuliani, Proteins as sponges: A statistical journey along protein structure organization principles, in: Journal of Chemical Information and Modeling, 2012: pp. 474–482. https://doi.org/10.1021/ci2005127.

[5] N. Arrigo, P. Paci, L. di Paola, D. Santoni, M. de Ruvo, A. Giuliani, F. Castiglione, Characterizing Protein Shape by a Volume Distribution Asymmetry Index, The Open Bioinformatics Journal. 6 (2012). https://doi.org/10.2174/1875036201206010020.

[6] A. di Venere, E. Nicolai, V. Minicozzi, A.M. Caccuri, L. di Paola, G. Mei, The Odd Faces of Oligomers: The Case of TRAF2-C, A Trimeric C-Terminal Domain of TNF Receptor-Associated Factor, International Journal of Molecular Sciences. 22 (2021) 5871. https://doi.org/10.3390/ijms22115871.

[7] C.S. de P. Perez, A. Zaccaria, T. di Matteo, Asymmetric Relatedness from Partial Correlation, Entropy. 24 (2022). https://doi.org/10.3390/e24030365.

[8] A. de la Fuente, N. Bing, I. Hoeschele, P. Mendes, Discovery of meaningful associations in genomic data using partial correlation coefficients, Bioinformatics. 20 (2004). https://doi.org/10.1093/bioinformatics/bth445.
